# Supplementary material for: Heterogeneous associations of a mobile health-based disease management program on uncontrolled hypertension: A target trial emulation study
Source: PLOS Digit Health. 2026 Mar 5;5(3):e0001268. doi: 10.1371/journal.pdig.0001268 (PMC12962524; doi:10.1371/journal.pdig.0001268)
Supplement: S1 Fig — (DOCX) [file pdig.0001268.s001.docx]

**S1 Fig. The empirical distribution of estimated treatment probabilities.**


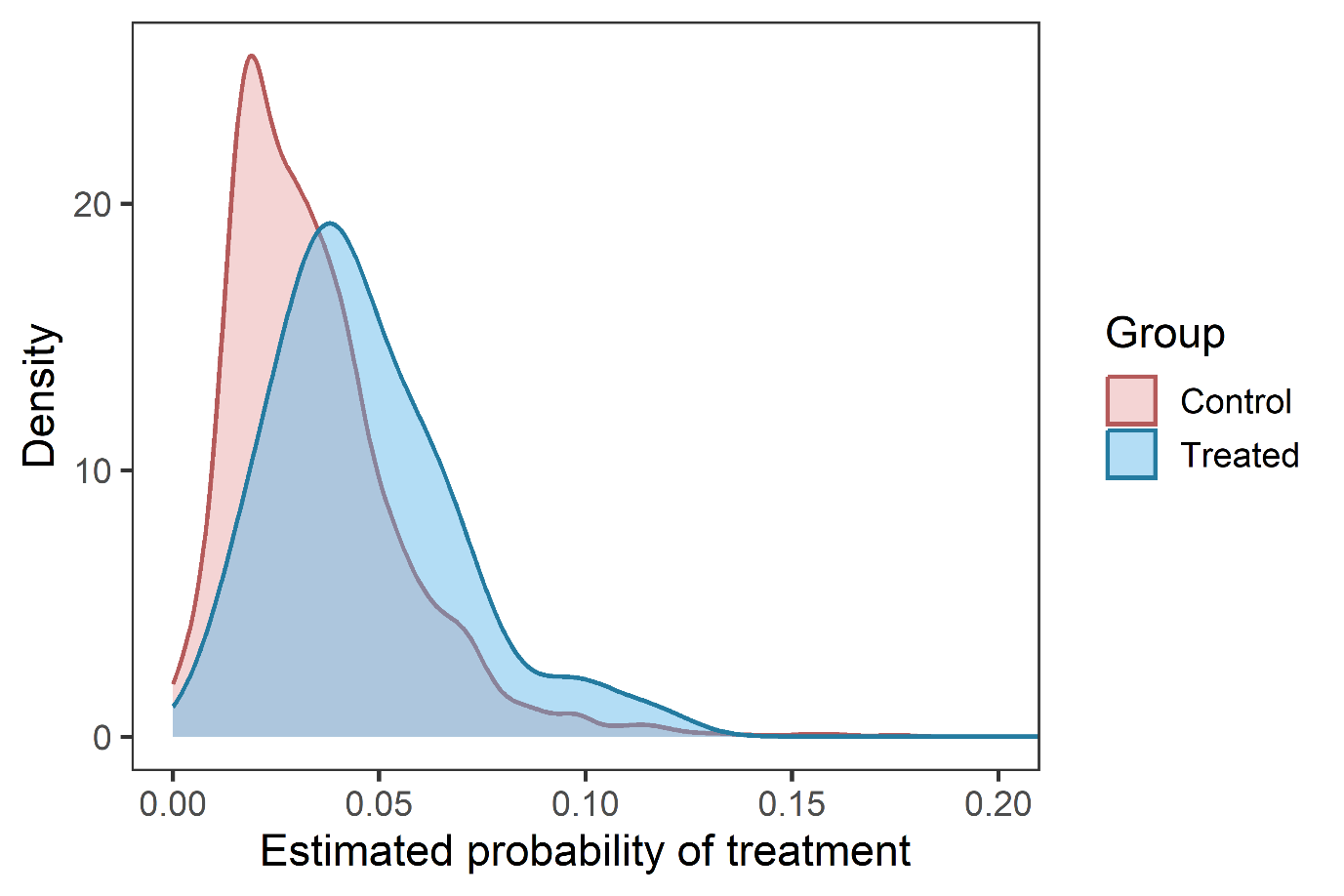


Estimated treatment probabilities were obtained from a logistic regression model including baseline covariates as predictors of participation in the mHealth-based disease management program.
