# Supplementary material for: Heterogeneous associations of a mobile health-based disease management program on uncontrolled hypertension: A target trial emulation study
Source: PLOS Digit Health. 2026 Mar 5;5(3):e0001268. doi: 10.1371/journal.pdig.0001268 (PMC12962524; doi:10.1371/journal.pdig.0001268)
Supplement: S1 Table — (DOCX) [file pdig.0001268.s002.docx]

**S1 Table. Cluster effects and the differences in baseline characteristics between individuals with high vs. low individual treatment effect (Secondary outcome = difference in *diastolic* blood pressure between baseline and follow-up).**

| Clusters | CATE (95% CIs) | Proportion Without Intention to Improve Lifestyle Habits | Proportion of Current Smokers | Diastolic blood pressure (mean) | Proportion of Non-Drinkers or Rare Drinkers | Glutamate Oxaloacetate Transaminase (mean) | Proportion of Not Engaging in Walking | Gamma-GTP (mean) | Proportion of Not Engaging in Exercise that Causes Light sweating (≥30 min) | Age (mean) | Proportion of Occasional Drinkers |
| --- | --- | --- | --- | --- | --- | --- | --- | --- | --- | --- | --- |
| High-benefit | −11.12 (−11.52 to −10.72) | 0.16 | 0.75 | 81.14 | 0.39 | 25.18 | 0.50 | 45.25 | 0.83 | 48.46 | 0.29 |
| Low-benefit | 5.52 (5.14 to 5.90) | 0.17 | 0.82 | 80.57 | 0.52 | 28.15 | 0.53 | 55.46 | 0.79 | 51.39 | 0.28 |

CATE, conditional average treatment effect; CI, confidence interval.
