# Supplementary material for: Heterogeneous associations of a mobile health-based disease management program on uncontrolled hypertension: A target trial emulation study
Source: PLOS Digit Health. 2026 Mar 5;5(3):e0001268. doi: 10.1371/journal.pdig.0001268 (PMC12962524; doi:10.1371/journal.pdig.0001268)
Supplement: S2 Table — (DOCX) [file pdig.0001268.s003.docx]

**S2 Table. Cluster effects and the differences in baseline characteristics between individuals with high vs. low individual treatment effect (Secondary outcome = difference in *systolic* blood pressure between baseline and follow-up).**

| Clusters | CATE (95% CIs) | Proportion Without Intention to Improve Lifestyle Habits | Proportion of Current Smokers | Diastolic blood pressure (mean) | Proportion of Non-Drinkers or Rare Drinkers | Glutamate Oxaloacetate Transaminase (mean) | Proportion of Not Engaging in Walking | Gamma-GTP (mean) | Proportion of Not Engaging in Exercise that Causes Light sweating (≥30 min) | Age (mean) | Proportion of Occasional Drinkers |
| --- | --- | --- | --- | --- | --- | --- | --- | --- | --- | --- | --- |
| High-benefit | −8.34 (−8.85 to −7.83) | 0.10 | 0.70 | 81.98 | 0.25 | 25.80 | 0.47 | 47.74 | 0.83 | 49.20 | 0.32 |
| Low-benefit | 5.38 (5.09–5.68) | 0.31 | 0.88 | 79.76 | 0.66 | 27.17 | 0.55 | 52.06 | 0.79 | 50.89 | 0.26 |

CATE, conditional average treatment effect; CI, confidence interval.
