# Supplementary material for: Heterogeneous associations of a mobile health-based disease management program on uncontrolled hypertension: A target trial emulation study
Source: PLOS Digit Health. 2026 Mar 5;5(3):e0001268. doi: 10.1371/journal.pdig.0001268 (PMC12962524; doi:10.1371/journal.pdig.0001268)
Supplement: S3 Table — (DOCX) [file pdig.0001268.s004.docx]

**S3 Table. Sensitivity analysis with the variable importance threshold set to 0.20**

| Clusters | CATE | Proportion Without Intention to Improve Lifestyle Habits | Proportion of Current Smokers | Diastolic blood pressure (mean) | Proportion of Non-Drinkers or Rare Drinkers | Glutamate Oxaloacetate Transaminase (mean) |
| --- | --- | --- | --- | --- | --- | --- |
| High-benefit | −0.17 | 0.08 | 0.71 | 82.91 | 0.39 | 24.70 |
| Low-benefit | 0.10 | 0.28 | 0.90 | 77.98 | 0.56 | 28.98 |

| Clusters | Proportion of Non-Drinkers or Rare Drinkers | Gamma-GTP (mean) | Proportion of Not Engaging in Exercise that Causes Light sweating (≥30 min) | Age (mean) | Proportion of Occasional Drinkers | HDL cholesterol (mean) | Glutamate Pyruvate Transaminase (mean) |
| --- | --- | --- | --- | --- | --- | --- | --- |
| High-benefit | 0.39 | 40.64 | 0.85 | 49.15 | 0.34 | 59.51 | 31.24 |
| Low-benefit | 0.56 | 62.36 | 0.75 | 51.35 | 0.22 | 63.15 | 33.63 |

CATE, conditional average treatment effect
