# Supplementary material for: Heterogeneous associations of a mobile health-based disease management program on uncontrolled hypertension: A target trial emulation study
Source: PLOS Digit Health. 2026 Mar 5;5(3):e0001268. doi: 10.1371/journal.pdig.0001268 (PMC12962524; doi:10.1371/journal.pdig.0001268)
Supplement: Attachment — Submitted filename: Mystar_TTE_Response.docx [file pdig.0001268.s007.docx]

**Response to Editor and Reviewers**

We sincerely thank the Reviewers and Editor for their thoughtful and constructive feedback. We have carefully revised our manuscript in accordance with the comments provided. Below we provide point-by-point responses to each comment. All modifications to the manuscript are highlighted and noted with track changes.

**Review Comments to the Author**

***Reviewer #1: 1. The manuscript numbers are rather specific to the submission process for the journal, which is fine, but the Short Title is duplicated. Ensure that it appears only once or as necessary by the final layout.***

RESPONSE:

We appreciate the reviewer’s careful attention to formatting. We have confirmed that the short title (“Heterogeneous Effects of mHealth for Hypertension”) appears only once in the revised manuscript and does not duplicate the main title in the final layout.

***2.The character of the "low-benefit cluster" (CATE of 0.10) is presented without a deep interpretive understanding. That the intervention increased this group's risk of uncontrolled hypertension by 10% needs to be talked about with care, particularly in the context of traditional treatment alone.***

***The finding that the high-benefit group walked but didn't engage in light sweating exercise may be deceptive. This is a distinction which must be clarified in the Discussion—is the group receptive to low-barrier strategies, or is program design beneficial for those with an intermediate activity baseline?.***

***The accepted limitation of relying only on baseline characteristics to assess ITE, not counting changes in health behaviors or program participation during the six-month intervention, is a significant limitation for discerning heterogeneity fully.***

***In the Discussion, devote a paragraph to interpreting the CATE of 0.10 for the low-benefit cluster. An affirmative CATE signifies increased risk of uncontrolled hypertension. Provide a conservative, evidence-based description. Is this possibly a population with such suboptimal adherence/comorbidities that low intensities of remote coaching are insufficient or disruptive to more pressing intense care (usual treatment)?. Is it possible that this population has problems (e.g., psychological, socioeconomic) not accounted for by baseline covariates and thus have poor outcomes regardless of the intervention?***

***It is suggested that the authors to include about AI/ML in decision support or healthcare optimization because the approach largely depends on machine learning (SuperLearner, clustering) to enhance decision-making (personalized treatment). The approach in the manuscript utilizes ensemble machine learning in estimating individual treatment effects, which is essentially an improvement and optimization problem of decision-making. The suggested paper from the reference: "Blockchain-Enabled Healthcare Optimization: Enhancing Security and Decision-Making Using the Mother Optimization Algorithm" this paper discusses utilizing advanced algorithms to improve decision-making and enable safe, transparent, and efficient medical data handling. This is in line most particularly with the mission of the manuscript to utilize ML for assisting with enhanced treatment choice.***

***The authors must react to the following main points: Low-Benefit Cluster Interpretation: Describe the interpretation of the CATE of 0.10 under Discussion. Provide a cautious, soundly argued reason why the intervention negatively affected this subgroup (e.g., condition complexity, non-adherence, low level of intervention). Clearness on Exercise: Clarify the definition of high-benefit cluster walking but not in light sweating exercise to clearly say the clinical/behavioral implication: more likely to be a change ready group and would benefit from low-to-moderate intensity digital nudges.***

RESPONSE: We appreciate this thoughtful and entirely valid comment regarding the interpretation of heterogeneous treatment effects and the implications of our machine-learning–based approach. We agree that both the positive CATE in the low-benefit cluster and the physical activity pattern characterizing the high-benefit cluster require cautious, clinically grounded interpretation. In response, we have (i) added a dedicated paragraph in the Discussion to more carefully interpret the CATE of 0.10 in the low-benefit cluster, (ii) clarified the behavioral and clinical implications of the high-benefit cluster’s walking but lack of moderate-to-vigorous exercise, (iii) slightly strengthened our description of the limitations of using only baseline characteristics to assess individual treatment effects, (iv) added a brief methodological remark on how our SuperLearner-based framework can support individualized decision-making in chronic disease management, and (v) related our findings to the recently proposed “high-benefit approach” in precision medicine, highlighting how data-driven estimation of heterogeneous treatment effects can help prioritize mHealth-based support for subpopulations with greater expected benefit while identifying those who may require alternative strategies. We hope these revisions address your concerns and provide a clearer and more balanced interpretation of the observed heterogeneity.

Discussion (lines 356-364)

In contrast, participants in the low-benefit cluster exhibited a higher probability of uncontrolled hypertension (CATE = 0.10). This positive CATE should not be interpreted as evidence that the program was harmful, but rather as indicating a subgroup with limited responsiveness to low-intensity, app-based coaching. Individuals in this cluster may have more complex comorbidity profiles, lower adherence to lifestyle recommendations or antihypertensive treatment, or psychosocial and socioeconomic challenges that were not fully captured by baseline covariates. For such patients, more intensive, multimodal, or face-to-face support beyond the present mHealth-based program may be required to achieve meaningful improvements in blood pressure control.

Discussion (lines 339-345)

Similarly, participants who walk regularly but do not engage in moderate-to-vigorous exercise may represent an intermediate activity level, where incremental behavior changes lead to measurable benefits [37]. Taken together, these patterns suggest that the high-benefit cluster represents individuals with an intermediate activity baseline who are particularly receptive to low-barrier strategies, such as app-based monitoring and step-goal prompts, rather than prescriptions for vigorous exercise.

Discussion (lines 397-401)

From a methodological perspective, this framework illustrates how ensemble machine learning can enhance individualized decision-making in chronic disease management. Integrating causal inference with data-adaptive modeling offers a transparent and scalable pathway for optimizing personalized digital health interventions.

Discussion (lines 367-372)

Consistent with the recently proposed high-benefit approach in precision medicine, which emphasizes targeting individuals who are most likely to benefit rather than those who are merely at highest risk [39], our findings illustrate how data-driven estimation of heterogeneous treatment effects can help prioritize mHealth-based support for subpopulations with greater expected gains while also identifying groups with limited benefit who may require alternative intervention strategies.

***Reviewer #2: Kanai and colleagues explored the heterogeneous effects of a mobile health-based disease management program on uncontrolled hypertension. The research topic is interesting, but the findings are undermined by the study design.***

***Major comments:***

***Major concerns come from the huge disparities in baseline characteristics of the study participants between Control group and Treated group (Table 2). Key confounders such as sex, diabetes, age, triglycerides and so on are imbalanced between the two groups. The Treated group mostly has more severe disease conditions than the Control group, making the target trial emulation not reliable.***

RESPONSE: We thank the reviewer for this important comment. We agree that baseline characteristics differed substantially between the treated and control groups (Table 2), and such imbalance raises valid concerns regarding the identifiability of causal effects. Target trial emulation does not require baseline comparability, but it does require adequate adjustment for confounding and sufficient empirical support for both treatment strategies. In our study, we used the G-formula with a data-adaptive SuperLearner to flexibly model the conditional outcome regression and standardize over the joint distribution of measured covariates. This approach adjusts for baseline differences—such as age, sex, metabolic risk factors, and comorbidities—thereby addressing measured confounding without relying on propensity score weighting.

In response to the reviewer’s concern about the magnitude of imbalance, we further examined the empirical distribution of estimated treatment probabilities (Supplementary Figure 1). Consistent with the selective uptake of the intervention, the treated group demonstrated higher estimated probabilities than the control group, and overall overlap was limited. Nonetheless, the density plot shows a clear non-zero region of common support between approximately 0.02 and 0.06, indicating that empirical positivity was restricted but not violated. We now explicitly acknowledge in the Discussion that both limited overlap and the potential for residual confounding remain inherent limitations of observational analyses.

Discussion (lines 420-436)

Fourth, individuals with chronic complications may exhibit lower adherence to both therapeutic regimens and mHealth-based interventions, potentially leading to an underestimation of the observed intervention effects. Fifth, baseline characteristics differed substantially between the treated and untreated groups, raising concerns about the identifiability of causal effects. Although the G-computation approach adjusts for measured confounders through model-based standardization, its validity depends on the positivity assumption. To evaluate this, we examined the empirical distribution of estimated treatment probabilities and observed a limited but clearly non-zero region of overlap between groups (Supplementary Figure 1). This indicates that empirical positivity was restricted but not violated. Finally, the potential for unmeasured confounding remains, despite the application of robust causal inference techniques. In particular, information on medication use, adherence patterns, and socioeconomic or behavioral factors, such as digital literacy and access to technology, was not available and could not be adjusted for. Furthermore, several baseline differences between groups may have partially influenced the observed associations. We addressed this potential confounding using G-computation to obtain marginal estimates adjusted for baseline covariates, and our calculated E-values indicated moderate robustness to unmeasured confounding [46].

***In Table 1, the Inclusion criteria are ambiguous. If the authors aim to explore the impact of mHealth on hypertension, they should focus on those with hypertension undergoing drug therapies. It is unclear why the authors equally included the patients with diabetes and dyslipidaemia. The authors also excluded patients with “severe” cardiac diseases such as arrhythmias, though arrhythmias such as atrial fibrillation are quite common in those patients with hypertension. It is not quite clear what is “severe”.***

RESPONSE: We thank the reviewer for this insightful comment regarding the inclusion criteria. The eligibility criteria were defined to reflect the actual enrollment policy of the original mHealth-based disease management program. This program targeted adults with common lifestyle-related diseases, including hypertension, diabetes, and dyslipidemia, who were either receiving pharmacological treatment or had a history of coronary artery disease or stroke. In other words, individuals were identified as having an elevated risk of future cardiovascular events based on their existing diagnoses and treatments. Because these conditions frequently coexist and share overlapping pathophysiological and behavioral risk factors, the program was implemented as a disease management program for lifestyle-related conditions, with the aim of supporting integrated management and preventing progression of cardiovascular complications, rather than as a single-disease intervention.

While the program itself enrolled patients with hypertension, diabetes, or dyslipidemia, the present analysis focuses specifically on blood pressure control among participants with a diagnosis of hypertension. Hypertension was the only condition used to define the primary outcome (uncontrolled hypertension), whereas diabetes and dyslipidemia were treated as coexisting lifestyle-related conditions that characterize the real-world population enrolled in the mHealth-based disease management program used for our target trial emulation.

Regarding the exclusion of “severe cardiac diseases,” we apologize that the GitHub link provided in the original submission was not accessible. We have corrected the URL in the revised manuscript so that the operational definition of these exclusion criteria can be verified (<https://github.com/PREVENT-Inc/MyscopeMasterList/tree/master/Out_of_scope>). In brief, this category was intended to capture clinically unstable cardiac conditions and related comorbidities for which intensive lifestyle modification and remote coaching might be unsafe or difficult to adhere to, such as symptomatic atrial fibrillation or other tachyarrhythmias requiring close monitoring, dilated or hypertrophic cardiomyopathy, or other unstable cardiac diseases. In addition, participation in the mHealth-based disease management program required approval from each participant’s primary physician, which served as an additional safeguard to ensure that only those for whom the intervention was considered clinically appropriate were enrolled. We believe that these screening procedures, together with the explicit exclusion criteria, helped to promote both the safety and feasibility of the program for patients at elevated cardiovascular risk.

Methods (lines 114-125)

This study included participants diagnosed with hypertension, diabetes, or dyslipidemia who were either receiving medication or had a history of coronary artery disease or stroke. These eligibility criteria reflected the actual enrollment policy of the original mHealth-based disease management program, which targeted people with common lifestyle-related diseases rather than hypertension alone. Because these conditions frequently coexist and share overlapping pathophysiological and behavioral risk factors, the program was implemented as a disease management program for lifestyle-related conditions, with the aim of supporting integrated management and preventing progression of cardiovascular complications, rather than as a single-disease intervention. Eligible individuals were identified through the Specific Health Checkup and employer-provided medical claims data and were invited to participate in the mHealth-based disease management program.

Methods (lines 126-140)

Exclusion criteria included participants with medical conditions that could interfere with participation, such as severe cardiac diseases (e.g., symptomatic arrhythmias requiring close monitoring, dilated and hypertrophic cardiomyopathy, and other unstable cardiac conditions), end-stage kidney disease, or mental disorders; individuals taking medications such as cardiotonic agents, immunosuppressants, or antipsychotics; and those deemed unsuitable by the health insurance association based on predefined criteria.These exclusion criteria were operationalized using predefined contraindication codes for the remote, app-based disease management program and were intended to identify individuals for whom safe and stable participation, including adequate adherence, might be difficult. A complete list of exclusionary diagnoses is available at the GitHub link (<https://github.com/PREVENT-Inc/MyscopeMasterList/tree/master/Out_of_scope>). Additionally, participants with follow-up periods of less than 275 days were excluded, accounting for the time from invitation to the completion of the six-month intervention.

***The study Outcome of uncontrolled hypertension may be better replaced by actual blood pressure values or the difference in blood pressure between the baseline and follow-up.***

RESPONSE: We appreciate the reviewer’s insightful comment. In response, we conducted a reanalysis and defined these variables as secondary outcomes, representing the differences in systolic and diastolic blood pressure between baseline and follow-up. Furthermore, we now briefly refer to these secondary results in the Discussion to contextualize the observed 5.2% absolute reduction in uncontrolled hypertension in terms of continuous blood pressure changes. We hope that these additions address the reviewer’s concern.

Methods (lines 186-187)

The secondary outcomes were the differences in systolic and diastolic blood pressure between baseline and follow-up.

Results (lines 262-264)

In addition, participation in the program was associated with a mean change in systolic blood pressure of −1.22 mmHg (95% CI: −1.58 to −0.87) and in diastolic blood pressure of −3.67 mmHg (95% CI: −4.14 to −3.22).

Results (lines 287-293)

Supplementary Tables 1 and 2 present the results of our secondary outcomes, comparing the baseline characteristics of the high-benefit and low-benefit clusters based on the covariates selected in the primary outcome analysis. For the secondary outcome analysis, the mean CATE for systolic blood pressure was −8.34 mmHg (95% CI: −8.85 to −7.83) in the high-benefit cluster and 5.38 mmHg (95% CI: 5.09 to 5.68) in the low-benefit cluster. For diastolic blood pressure, the mean CATE was −11.12 mmHg (95% CI: −11.52 to −10.72) in the high-benefit cluster and 5.52 mmHg (95% CI: 5.14 to 5.90) in the low-benefit cluster.

Discussion (lines 316-327)

While minimal clinically important differences for blood pressure reduction in digital interventions remain unclear, our study employed a pragmatic trial design using uncontrolled hypertension—defined as systolic ≥140 mmHg or diastolic ≥90 mmHg—as a clinically meaningful endpoint. Based on this definition, participation in the mHealth-based program resulted in a 5.2% absolute reduction in the prevalence of uncontrolled hypertension compared with conventional treatment. This approach enhances the real-world applicability of our findings and aligns the evaluation of digital interventions with established clinical thresholds. Consistent with this threshold-based endpoint, secondary analyses of continuous blood pressure values showed mean changes in systolic and diastolic blood pressure (−1.22 and −3.67 mmHg, respectively). These mean reductions are compatible with the observed decrease in uncontrolled hypertension and should be interpreted alongside the substantial heterogeneity in individual treatment responses described below.

Discussion (lines 388-396)

A key strength of this study lies in its use of a three-step, data-adaptive framework that integrates outcome regression via the G-formula with SuperLearner-based model specification to estimate treatment heterogeneity. By combining the G-formula with a SuperLearner ensemble model, we can assess individual-level treatment effects while minimizing overfitting and preserving interpretability. This approach extends beyond traditional ATE estimates, providing a more nuanced understanding of how different subgroups respond to mHealth-based interventions [45]. Moreover, the consistent direction of effects between the primary binary endpoint and the secondary continuous outcomes supports the robustness of the estimated heterogeneous treatment effects.

***Blood pressure was assessed annually during the health checkup. Is it only one measurement? How was it performed? How is the reliability of this measurement if it was only measured once?***

RESPONSE: We appreciate the reviewer’s comment regarding the reliability of blood pressure measurement. In this study, blood pressure was assessed annually during the Specific Health Checkup. Blood pressure was measured twice using a standard sphygmomanometer or automated device on the right arm after a 5-minute rest in the sitting position, and the average of the two readings was recorded. This procedure follows the standard protocol of Japan’s Specific Health Checkup program, which has been widely used in large-scale epidemiological studies published internationally (e.g., Tamaki et al., Clin Kidney J, 2024; Tsushita et al., J Atheroscler Thromb, 2018). We have added the following explanations in the Method sections:

Methods (lines 187-193)

Blood pressure was assessed annually during the Specific Health Checkup. In principle, blood pressure was measured twice using a standard sphygmomanometer or automated device on the right arm after a 5-minute rest in the sitting position, and the average of the two readings was recorded. This procedure follows the standard protocol of Japan’s Specific Health Checkup program, which has been widely used in large-scale epidemiological studies published internationally [31,32].

***Minor comments:***

***In the Treatment arm, did patients follow all three programs?***

RESPONSE:

Thank you very much for this helpful question. In our study, there was one integrated mHealth-based disease management program in the treatment arm. The three items listed in Table 1 refer to the core components of this single program, not to three separate programs. All participants in the treatment arm received conventional treatment plus this integrated mHealth-based program. To avoid misunderstanding, we have revised the wording in Table 1 to clarify that these items represent key components of one mHealth-based disease management program.

Table 1

[Key components of the mobile health-based disease management program]

(1)	12 phone sessions over six-months with healthcare professionals, plus chat support

(2)	Use of a mobile app to track lifestyle data and review educational content

(3)	Personalized coaching and health goal-setting focused on behavior change

***The authors said that they followed the STROBE guideline, so a checklist should be provided.***

RESPONSE: Thank you for this important remark. We have now provided the completed STROBE checklist as a supplementary file.

***In “Inclusion and exclusion criteria” section, the Guithub link for the exclusionary diagnoses is not accessible. The authors may check it.***

RESPONSE:

Thank you very much for pointing this out. The GitHub link provided in the original submission contained an error in the path. We have corrected the URL in the “Inclusion and exclusion criteria” section of the Methods so that it now directs to the appropriate repository for the exclusionary diagnoses (<https://github.com/PREVENT-Inc/MyscopeMasterList/tree/master/Out_of_scope>), and we have confirmed that it is accessible.

***Reviewer #3: - The paper claims that participation in a mobile health–based disease management program was associated with a lower prevalence of uncontrolled hypertension after one year, and that this effect varied across individuals. It applies target trial emulation with causal machine learning to estimate both average and individual treatment effects.***

***These claims are important and timely for digital health, as they promote rigorous causal methods and personalized intervention evaluation in real-world data. However, the causal inference remains moderate given the observational design and limited sensitivity analyses.***

***The claims are only partially novel. The same dataset and blood pressure thresholds were analyzed in (J Med Internet Res 2023;25:e43809) doi: 10.2196/43809, which already demonstrated improved BP control with the mHealth app. The current manuscript mainly reframes the same study under a target-trial-emulation approach and adds heterogeneity analysis using causal ML. Thus, it contributes methodologically rather than substantively and could be considered complementary research rather than fully original work.***

RESPONSE: We appreciate the reviewer’s thoughtful comment regarding the novelty of the present study. We would like to clarify that the current analysis was conducted using an independent cohort from a different country, distinct from that used in the previous publication that the reviewer indicated (J Med Internet Res 2023;25:e43809). Moreover, the research objectives and analytic framework differ substantially. As noted in the revised manuscript, systematic reviews and meta-analyses have indicated that most digital health studies have relatively short follow-up periods (3–12 months) and provide limited evidence on sustained effects. Building upon these gaps, the present study evaluates whether the benefits of mHealth interventions persist over an extended period and identifies subgroups that derive the greatest benefit. Accordingly, this study represents a methodologically and contextually distinct investigation, expanding the evidence base for mHealth-based hypertension management across different healthcare systems and populations. We have clarified this distinction in the revised Introduction.

Introduction (lines 76-77)

Moreover, evidence on their long-term effects remains limited, as most studies have follow-up periods of only 3–12 months [12,18–20,22].

*Line 118: website is not working*

RESPONSE:

Thank you very much for pointing this out. The GitHub link provided in the original submission contained an error in the path. We have corrected the URL in the “Inclusion and exclusion criteria” section of the Methods so that it now directs to the appropriate repository for the exclusionary diagnoses (<https://github.com/PREVENT-Inc/MyscopeMasterList/tree/master/Out_of_scope>), and we have confirmed that it is accessible.

*In Methods, Table 1 (“Treatment assignment”), replace “Individuals were randomly allocated following the eligible assessment” with “Treatment assignment was emulated analytically within the target trial framework.”*

RESPONSE: We thank the reviewer for pointing out this inaccuracy. After careful consideration, we have revised the description as follows:

Table 1

Individuals were nonrandomly assigned to the treatment arms described below. Randomization was emulated using the G-formula, with adjustment for baseline covariates.

***Covariate-balance diagnostics: To align with current Target Trial Emulation guidance, please demonstrate that the emulated treatment and control groups were comparable at baseline. Add a Supplementary Figure or table showing standardized mean differences (SMDs) for all baseline covariates before and after adjustment or weighting.***

RESPONSE: We thank the reviewer for this important comment. In this study, we used the G-formula (also known as the outcome regression approach) to estimate the marginal mean outcome under each treatment strategy. Unlike weighting-based approaches such as inverse probability weighting, the G-formula does not create a reweighted pseudo-population or explicitly balance baseline covariates between treatment groups. Instead, it models the conditional outcome given covariates and then standardizes predictions over the joint distribution of baseline covariates in the full study population. Accordingly, covariate-balance diagnostics such as standardized mean differences are not directly applicable within the G-formula framework.

To address the reviewer’s concern regarding empirical support and comparability, we added Supplementary Figure 1 showing the empirical distribution of estimated treatment probabilities. As expected given the selective uptake of the intervention, the treated group exhibited higher estimated probabilities than the untreated group, resulting in limited overall overlap. Nevertheless, a clear non-zero region of common support was present, indicating that empirical positivity was restricted but not violated. In addition, Table 2 already reports baseline characteristics across treatment groups, illustrating the degree of initial imbalance We have added corresponding clarifications in the Methods and Discussion sections.

Methods (lines 199-202)

First, we estimated the ITE of the mHealth-based disease management program program on the outcome using the G-formula (outcome model), implemented with data-adaptive model specification via the SuperLearner ensemble machine learning algorithm [33].

Discussion (lines 420-436)

Fourth, individuals with chronic complications may exhibit lower adherence to both therapeutic regimens and mHealth-based interventions, potentially leading to an underestimation of the observed intervention effects. Fifth, baseline characteristics differed substantially between the treated and untreated groups, raising concerns about the identifiability of causal effects. Although the G-computation approach adjusts for measured confounders through model-based standardization, its validity depends on the positivity assumption. To evaluate this, we examined the empirical distribution of estimated treatment probabilities and observed a limited but clearly non-zero region of overlap between groups (Supplementary Figure 1). This indicates that empirical positivity was restricted but not violated. Finally, the potential for unmeasured confounding remains, despite the application of robust causal inference techniques. In particular, information on medication use, adherence patterns, and socioeconomic or behavioral factors, such as digital literacy and access to technology, was not available and could not be adjusted for. Furthermore, several baseline differences between groups may have partially influenced the observed associations. We addressed this potential confounding using G-computation to obtain marginal estimates adjusted for baseline covariates, and our calculated E-values indicated moderate robustness to unmeasured confounding [46].

***Sensitivity analyses for unmeasured confounding: Please add a short subsection titled “Sensitivity analyses” in the Methods and present corresponding results. Mention in the Discussion how sensitive or robust the main findings are to moderate unmeasured confounding. This addition is required to support the validity of the causal interpretation.***

RESPONSE: We appreciate this important suggestion. We have added a new subsection titled “Sensitivity analyses” in the Methods section to describe the approach used to assess the potential impact of unmeasured confounding. Specifically, we conducted sensitivity analyses using the E-value framework, which quantifies the minimum strength of association that an unmeasured confounder would need to have with both the exposure and the outcome to fully explain away the observed effect estimate.In the Discussion, we have also added a statement clarifying that the main findings were robust to moderate unmeasured confounding, as the corresponding E-values were substantially larger than the observed associations between known covariates and the outcome.

Methods (lines 226-232)

Sensitivity analyses

To assess the robustness of the variable selection process, we additionally conducted two types of sensitivity analyses. First, we used alternative thresholds of 0.20 and 0.30 for defining potential effect modifiers. Second, we calculated E-values to evaluate the potential impact of unmeasured confounding, which quantify the minimum strength of association that an unmeasured confounder would need to have with both the exposure and the outcome to fully explain the observed associations [36].

Results(lines 257-264)

For the ATE of the mHealth-based disease management program on uncontrolled hypertension, participation in the program was associated with a 5.2% (95% confidence interval [CI]: 4.4%–6.0%) lower risk of uncontrolled hypertension, corresponding to a risk ratio of 0.81 (95% CI: 0.78 to 0.84) compared with conventional treatment alone. This absolute risk reduction corresponds to an NNT of 19.2 (95% CI: 16.7 to 22.7) over the study’s follow-up period. The calculated E-value for this association was 1.77. In addition, the program led to reductions in systolic blood pressure by −1.22 mmHg (95% CI: −1.58 to −0.87) and in diastolic blood pressure by −3.67 mmHg (95% CI: −4.14 to −3.22).

Results (lines 281-286)

In the high-benefit cluster, the mean CATE was −0.17 (95% CI: −0.19 to −0.15), while in the low-benefit cluster, the mean CATE was 0.10 (95%CI: 0.34 to 0.48) for the high-benefit cluster and 1.35 (95% CI: 1.28 to 1.42) for the low-benefit cluster. The absolute risk reduction for the high-benefit cluster corresponds to an NNT of 5.9 (95% CI, 5.3 to 6.7) over the study’s follow-up period. The E-values for these estimates were 4.31 and 2.04, respectively.

Discussion (lines 226-232)

Finally, the potential for unmeasured confounding remains, despite the application of robust causal inference techniques. In particular, information on medication use, adherence patterns, and socioeconomic or behavioral factors, such as digital literacy and access to technology, was not available and could not be adjusted for. Furthermore, several baseline differences between groups may have partially influenced the observed associations. We addressed this potential confounding using G-computation to obtain marginal estimates adjusted for baseline covariates, and our calculated E-values indicated moderate robustness to unmeasured confounding [46].

***The main outcome is currently binary (“uncontrolled hypertension” yes/no). To enhance clinical interpretability, please also report continuous BP outcomes (ΔSBP and ΔDBP in mmHg, mean ± SD and 95 % CI).***

RESPONSE: We appreciate the reviewer’s insightful comment. In response, we conducted a reanalysis and defined these variables as secondary outcomes, representing the differences in systolic and diastolic blood pressure between baseline and follow-up. Furthermore, we now briefly refer to these secondary results in the Discussion to contextualize the observed 5.2% absolute reduction in uncontrolled hypertension in terms of continuous blood pressure changes. We hope that these additions address the reviewer’s concern.

Methods (lines 186-187)

The secondary outcomes were the differences in systolic and diastolic blood pressure between baseline and follow-up.

Results (lines 262-264)

In addition, participation in the program was associated with a mean change in systolic blood pressure of −1.22 mmHg (95% CI: −1.58 to −0.87) and in diastolic blood pressure of −3.67 mmHg (95% CI: −4.14 to −3.22).

Results (lines 287-293)

Supplementary Tables 1 and 2 present the results of our secondary outcomes, comparing the baseline characteristics of the high-benefit and low-benefit clusters based on the covariates selected in the primary outcome analysis. For the secondary outcome analysis, the mean CATE for systolic blood pressure was −8.34 mmHg (95% CI: −8.85 to −7.83) in the high-benefit cluster and 5.38 mmHg (95% CI: 5.09 to 5.68) in the low-benefit cluster. For diastolic blood pressure, the mean CATE was −11.12 mmHg (95% CI: −11.52 to −10.72) in the high-benefit cluster and 5.52 mmHg (95% CI: 5.14 to 5.90) in the low-benefit cluster.

Discussion (lines 316-327)

While minimal clinically important differences for blood pressure reduction in digital interventions remain unclear, our study employed a pragmatic trial design using uncontrolled hypertension—defined as systolic ≥140 mmHg or diastolic ≥90 mmHg—as a clinically meaningful endpoint. Based on this definition, participation in the mHealth-based program resulted in a 5.2% absolute reduction in the prevalence of uncontrolled hypertension compared with conventional treatment. This approach enhances the real-world applicability of our findings and aligns the evaluation of digital interventions with established clinical thresholds. Consistent with this threshold-based endpoint, secondary analyses of continuous blood pressure values showed mean changes in systolic and diastolic blood pressure (−1.22 and −3.67 mmHg, respectively). These mean reductions are compatible with the observed decrease in uncontrolled hypertension and should be interpreted alongside the substantial heterogeneity in individual treatment responses described below.

Discussion (lines 388-396)

A key strength of this study lies in its use of a three-step, data-adaptive framework that integrates outcome regression via the G-formula with SuperLearner-based model specification to estimate treatment heterogeneity. By combining the G-formula with a SuperLearner ensemble model, we can assess individual-level treatment effects while minimizing overfitting and preserving interpretability. This approach extends beyond traditional ATE estimates, providing a more nuanced understanding of how different subgroups respond to mHealth-based interventions [45]. Moreover, the consistent direction of effects between the primary binary endpoint and the secondary continuous outcomes supports the robustness of the estimated heterogeneous treatment effects.

***Missing covariates: number of medications, adherence patterns, socioeconomic and behavioral factors (include digital literacy/technology access)***

RESPONSE: We thank the reviewer for this helpful comment. We agree that important covariates such as the number of medications, adherence patterns, and socioeconomic or behavioral factors—including digital literacy and access to technology—were not available in our dataset. We have now acknowledged this limitation in the revised manuscript as follows:

Discussion (lines 429-436)

Finally, as with all observational studies, the potential for unmeasured confounding remains, despite the application of robust causal inference techniques. In particular, information on medication use, adherence patterns, and socioeconomic or behavioral factors, such as digital literacy and access to technology, was not available and could not be adjusted for. Furthermore, several baseline differences between groups may have partially influenced the observed associations. We addressed this potential confounding using G-computation to obtain marginal estimates adjusted for baseline covariates, and our calculated E-values indicated moderate robustness to unmeasured confounding [46].

***Table 3: The reported CATE values (−0.17 vs 0.10) lack measures of precision (e.g., 95 % CIs or standard errors). The predefined variable importance threshold of 0.25 appears arbitrary. Please justify the choice or, alternatively, present sensitivity analyses using different thresholds (e.g., 0.20, 0.30) to demonstrate robustness.***

RESPONSE: We appreciate this valuable suggestion. In the revised version, we have added the 95% confidence intervals for the mean CATE values to provide measures of precision. Furthermore, to address the concern regarding the predefined threshold for variable importance, we conducted additional sensitivity analyses using alternative thresholds of 0.20 and 0.30. The results were consistent with the main analysis, indicating that our findings were robust to reasonable variations in the threshold selection. These revisions have been incorporated into the Results section and Supplementary Tables 3 and 4.

Methods (lines 226-232)

Sensitivity analyses

To assess the robustness of the variable selection process, we additionally conducted two types of sensitivity analyses. First, we used alternative thresholds of 0.20 and 0.30 for defining potential effect modifiers. Second, we calculated E-values to evaluate the potential impact of unmeasured confounding, which quantify the minimum strength of association that an unmeasured confounder would need to have with both the exposure and the outcome to fully explain the observed associations [36].

Results (lines 281-282)

In the high-benefit cluster, the mean CATE was −0.17 (95% CI: −0.19 to −0.15), while in the low-benefit cluster, the mean CATE was 0.10 (95% CI: 0.08 to 0.12).

***The Discussion (line 252-272) nicely interprets the observed heterogeneity in treatment effects in terms of behavioral readiness and motivation. While the discussion highlights behavioral readiness and engagement capacity, it would be useful to also acknowledge that these same factors might contribute to self-selection into higher adherence groups, potentially inflating apparent heterogeneity.***

RESPONSE: We thank the reviewer for this important point. We agree that behavioral readiness and engagement capacity may influence both responsiveness to the intervention and the likelihood of sustained adherence, potentially exaggerating the observed heterogeneity. In the revised Discussion, we now explicitly acknowledge that the characteristics defining the high-benefit cluster may also be associated with greater engagement with the app and coaching, and that our heterogeneity analysis, which is based on baseline covariates and does not incorporate time-varying adherence or behavior change, may therefore partially reflect self-selection into higher-adherence patterns. We believe this addition offers a more cautious and balanced interpretation of the heterogeneous treatment effe

Discussion (lines 349-355)

Nonetheless, the same behavioral readiness and engagement capacity that characterize the high-benefit cluster may also predispose these individuals to greater adherence to the mHealth program (e.g., more consistent app use and responsiveness to coaching). As our heterogeneity analysis relied solely on baseline covariates and did not explicitly model time-varying adherence or behavior change, part of the observed between-cluster differences in treatment effects may therefore reflect self-selection into higher-engagement patterns, in addition to genuine differences in causal responsiveness.

***Grammar and Readability:***

*Hyphenation: six-month, one-year, follow-up, data-adaptive, SuperLearner-based.*

RESPONSE: Thank you for this comment. We carefully re-checked the manuscript with specific attention to the use of “six months,” “one year,” “follow-up,” “data-adaptive,” and “SuperLearner-based.” We confirmed that these terms are used consistently and in an appropriate manner throughout the text. For example, we use forms such as “a one-year follow-up” when functioning as a compound modifier, whereas expressions like “the program lasted six months” or “over one year of follow-up” are intentionally left without hyphen as stand-alone time expressions. No substantial textual changes were required beyond this consistency check.

***Terms: uncontrolled hypertension (avoid “insufficient hypertension control”).***

RESPONSE: Thank you for this helpful comment. We have carefully revised the manuscript to ensure consistent use of terminology in line with your suggestion.

***Replace causal verbs in observational context: “was associated with” instead of “reduced,” “improved,” “caused.”***

RESPONSE: Thank you for this helpful comment. We have carefully revised the manuscript to ensure consistent use of terminology in line with your suggestion, replacing causal expressions with association-based wording where appropriate throughout the description of our observational findings.

***“modelling” → “modeling” (or choose one variant and use it throughout).***

RESPONSE: Thank you for pointing this out. We have standardized the spelling to “modeling” throughout the manuscript to ensure consistency.

***Line 35: was associated with a 5.2%***

RESPONSE: Thank you for pointing this out. We have revised it as you suggested.

***Covariates (Line 145): “engaging in moderate exercise (causing light sweating) …”;***

***“rare or non-drinkers” (instead of “merely or never”).***

RESPONSE: We appreciate this helpful suggestion. In response, we revised the description of physical activity to “engaging in exercise that causes light sweating” in the Covariates section and replaced “merely or never” with “rare or non-drinkers” in the table and related text to more clearly reflect the underlying questionnaire categories.

***Line 207: “the intervention reduced the risk…” → “was associated with a 5.2% lower risk/prevalence…”, same in Discussion first paragraph***

RESPONSE: Thank you for this helpful comment. We have revised the relevant sentences to avoid causal wording and to consistently use association-based expressions throughout the manuscript.

***Reviewer #4: The topic is relevant, the statistical design is robust, and the use of the G-formula method combined with the SuperLearner algorithm is innovative. The study benefits from being conducted within Japan’s well-structured health system, which provides equitable access to healthcare. However, several methodological aspects require clarification: there are statistically significant baseline differences between groups, and there is a bias related to lower adherence among patients with chronic or cardiac diseases. An individual-level analysis is needed to determine the weight and influence of each baseline variable on the final model.***

RESPONSE: We appreciate the reviewer’s insightful comment. We agree that baseline characteristics differed between groups, which could lead to confounding if analyzed using crude associations. To address this issue, we applied the G-formula to obtain marginal effect estimates standardized across baseline covariates rather than relying on simple associations. We also acknowledge the potential bias related to lower adherence among patients with chronic or cardiac diseases. Therefore, we calculated E-values to evaluate the robustness of our findings to potential unmeasured confounding.

Regarding the reviewer’s suggestion that an individual-level analysis is needed to determine the weight and influence of each baseline variable on the final model, we emphasize that our analysis already incorporated individual-level counterfactual predictions (ITEs) derived from the G-formula. We further assessed heterogeneity in these effects through cluster modeling based on ITEs and quantified the contribution of each covariate by comparing standardized z-scores across clusters. This approach enabled us to identify potential effect modifiers and to present variable importance measures that reflect each variable’s influence on individual-level treatment effects.

In addition, to evaluate empirical support for the treatment strategies, Supplementary Figure 1 shows the distribution of estimated treatment probabilities. As expected from the observed baseline differences, the treated group exhibited higher predicted probabilities, but a clear non-zero region of common support was present, indicating that empirical positivity was restricted but not violated.

We have revised the corresponding sections accordingly in the manuscript.

Methods (lines 229-232)

Second, we calculated E-values to evaluate the potential impact of unmeasured confounding, which quantify the minimum strength of association that an unmeasured confounder would need to have with both the exposure and the outcome to fully explain the observed associations [36].

Results (lines 257-264)

For the ATE of the mHealth-based disease management program on uncontrolled hypertension, participation in the program was associated with a 5.2% (95% confidence interval [CI]: 4.4%–6.0%) lower risk of uncontrolled hypertension, corresponding to a risk ratio of 0.81 (95% CI: 0.78 to 0.84) compared with conventional treatment alone. This absolute risk reduction corresponds to an NNT of 19.2 (95% CI: 16.7 to 22.7) over the study’s follow-up period. The calculated E-value for this association was 1.77. In addition, participation in the program was associated with a mean change in systolic blood pressure of −1.22 mmHg (95% CI: −1.58 to −0.87) and in diastolic blood pressure of −3.67 mmHg (95% CI: −4.14 to −3.22).

Results (lines 281-287)

In the high-benefit cluster, the mean CATE was −0.17 (95% CI: −0.19 to −0.15), while in the low-benefit cluster, the mean CATE was 0.10 (95% CI: 0.08 to 0.12). The corresponding risk ratios were 0.41 (95% CI: 0.34 to 0.48) for the high-benefit cluster and 1.35 (95% CI: 1.28 to 1.42) for the low-benefit cluster. The absolute risk reduction for the high-benefit cluster corresponds to an NNT of 5.9 (95% CI, 5.3 to 6.7) over the study’s follow-up period. The E-values for these estimates were 4.31 and 2.04, respectively.

Discussion (lines 420-436)

Fourth, individuals with chronic complications may exhibit lower adherence to both therapeutic regimens and mHealth-based interventions, potentially leading to an underestimation of the observed intervention effects. Fifth, baseline characteristics differed substantially between the treated and untreated groups, raising concerns about the identifiability of causal effects. Although the G-computation approach adjusts for measured confounders through model-based standardization, its validity depends on the positivity assumption. To evaluate this, we examined the empirical distribution of estimated treatment probabilities and observed a limited but clearly non-zero region of overlap between groups (Supplementary Figure 1). This indicates that empirical positivity was restricted but not violated. Finally, the potential for unmeasured confounding remains, despite the application of robust causal inference techniques. In particular, information on medication use, adherence patterns, and socioeconomic or behavioral factors, such as digital literacy and access to technology, was not available and could not be adjusted for. Furthermore, several baseline differences between groups may have partially influenced the observed associations. We addressed this potential confounding using G-computation to obtain marginal estimates adjusted for baseline covariates, and our calculated E-values indicated moderate robustness to unmeasured confounding [46].

***The writing is clear and well organized, though causal expressions such as “reduced” should be replaced with inferential phrasing like “was associated with.*”**

RESPONSE:

Thank you for this helpful comment. We have revised the relevant sentences to avoid causal wording and to consistently use association-based expressions throughout the manuscript.

***In the abstract and results, it should be emphasized that baseline differences, while small in magnitude, were statistically significant and may have influenced the findings, as the two groups represent populations with different baseline profiles.***

RESPONSE: We thank the reviewer for this helpful comment. While we agree that several baseline differences between groups may have partially influenced the observed associations, we believe this point is best addressed as a study limitation rather than in the Results or Abstract. We have therefore added the following statement to the Discussion section:

Discussion (lines 422-436)

Fifth, baseline characteristics differed substantially between the treated and untreated groups, raising concerns about the identifiability of causal effects. Although the G-computation approach adjusts for measured confounders through model-based standardization, its validity depends on the positivity assumption. To evaluate this, we examined the empirical distribution of estimated treatment probabilities and observed a limited but clearly non-zero region of overlap between groups (Supplementary Figure 1). This indicates that empirical positivity was restricted but not violated. Finally, the potential for unmeasured confounding remains, despite the application of robust causal inference techniques. In particular, information on medication use, adherence patterns, and socioeconomic or behavioral factors, such as digital literacy and access to technology, was not available and could not be adjusted for. Furthermore, several baseline differences between groups may have partially influenced the observed associations. We addressed this potential confounding using G-computation to obtain marginal estimates adjusted for baseline covariates, and our calculated E-values indicated moderate robustness to unmeasured confounding [46].

***The authors should also clarify that the assignment was voluntary and observational, not randomized, since the selection criteria and baseline imbalances (age, BMI, triglycerides, HDL, HbA1c, Charlson index) prevent the study from being considered experimental; these imbalances could bias effect estimation.***

RESPONSE: We thank the reviewer for this important comment. We agree that the present study was conducted within the framework of a target trial emulation, and not as an actual randomized trial. The treatment assignment was voluntary and observational, rather than randomized. The term target trial emulation refers to an analytical approach to observational data that aims to emulate the design and analysis of a hypothetical randomized trial, rather than implying that a trial was conducted. Because the two groups differed in several baseline characteristics (e.g., age, BMI, triglycerides, HDL, HbA1c, and Charlson index), we applied G-formula to obtain marginal effect estimates standardized for these covariates, thereby mitigating potential selection bias inherent in nonrandomized data.

Methods (lines 86-88)

In this study, we used “target trial emulation” framework, which emulates a target randomized control trial design, combined with a roadmap for causal inference to enhance the quality and interpretation of observational studies.

Table 1.

Individuals were nonrandomly assigned to the treatment arms described below. Randomization was emulated using G-formula, with adjustment for baseline covariates.

Discussion (lines 420-436)

Fourth, individuals with chronic complications may exhibit lower adherence to both therapeutic regimens and mHealth-based interventions, potentially leading to an underestimation of the observed intervention effects. Fifth, baseline characteristics differed substantially between the treated and untreated groups, raising concerns about the identifiability of causal effects. Although the G-computation approach adjusts for measured confounders through model-based standardization, its validity depends on the positivity assumption. To evaluate this, we examined the empirical distribution of estimated treatment probabilities and observed a limited but clearly non-zero region of overlap between groups (Supplementary Figure 1). This indicates that empirical positivity was restricted but not violated. Finally, the potential for unmeasured confounding remains, despite the application of robust causal inference techniques. In particular, information on medication use, adherence patterns, and socioeconomic or behavioral factors, such as digital literacy and access to technology, was not available and could not be adjusted for. Furthermore, several baseline differences between groups may have partially influenced the observed associations. We addressed this potential confounding using G-computation to obtain marginal estimates adjusted for baseline covariates, and our calculated E-values indicated moderate robustness to unmeasured confounding [46].

***A sensitivity or variable importance analysis (for example, variable importance plots, multivariable regression, or partial dependence analysis) is recommended to quantify the individual influence of each factor on the final model, along with stratified analyses by sex and the presence of cardiovascular disease.***

RESPONSE: We appreciate the reviewer’s valuable suggestion. Variable importance was estimated to quantify the relative contribution of each covariate to the estimated conditional average treatment effects. The results are presented as a variable importance plot (Figure 3), which highlights the covariates most strongly associated with treatment effect heterogeneity. Although stratified analyses by sex or cardiovascular disease status were not performed—because our primary aim was to explore heterogeneity across all covariates simultaneously rather than within specific subgroups—the variable importance results provide comparable insights into which factors most strongly influence the estimated treatment effects.

***It should be acknowledged that patients with chronic complications often show lower therapeutic and digital adherence, which could attenuate the observed effects.***

RESPONSE: We thank the reviewer for this important comment. We agree that patients with chronic complications may have lower adherence to both therapeutic regimens and digital interventions, which could attenuate the observed effects. We have now acknowledged this potential source of bias in the Discussion section as follows:

Discussion (lines 420-436)

Fourth, individuals with chronic complications may exhibit lower adherence to both therapeutic regimens and mHealth-based interventions, potentially leading to an underestimation of the observed intervention effects.

***The main clinical finding—an absolute reduction of 5.2%, or about 52 fewer uncontrolled cases per 1,000 treated patients (NNT ≈ 19)—should be expressed in clinical terms, while noting that adherence and selection biases may have contributed to the observed associations, as patients who comply with follow-up are also more likely to adhere to treatment, a correlated but non-causal phenomenon.***

RESPONSE: We thank the reviewer for this valuable suggestion. In response, we have now expressed the main clinical finding in absolute and clinically interpretable terms by including the number needed to treat (NNT) in the Results section. Furthermore, the Discussion now explicitly notes that behavioral readiness and engagement capacity may contribute to self-selection into higher-adherence patterns, so that part of the observed associations may reflect adherence and selection bias rather than purely causal effects.

Methods (lines 207-209)

In addition, the corresponding risk ratio was derived as the ratio of these probabilities. For interpretability, the number needed to treat (NNT) was calculated based on the absolute risk difference.

Results (lines 257-264)

For the ATE of the mHealth-based disease management program on uncontrolled hypertension, participation in the program was associated with a 5.2% (95% confidence interval [CI]: 4.4% to 6.0%) lower risk of uncontrolled hypertension, corresponding to a risk ratio of 0.81 (95% CI: 0.78 to 0.84) compared with conventional treatment alone. This absolute risk reduction corresponds to an NNT of 19.2 (95% CI: 16.7 to 22.7) over the study’s follow-up period.

Results (lines 282-286)

The corresponding risk ratios were 0.41 (95% CI: 0.34 to 0.48) for the high-benefit cluster and 1.35 (95% CI: 1.28 to 1.42) for the low-benefit cluster. The absolute risk reduction for the high-benefit cluster corresponds to an NNT of 5.9 (95% CI, 5.3 to 6.7) over the study’s follow-up period.

Discussion (lines 349-355)

Nonetheless, the same behavioral readiness and engagement capacity that characterize the high-benefit cluster may also predispose these individuals to greater adherence to the mHealth program (e.g., more consistent app use and responsiveness to coaching). As our heterogeneity analysis relied solely on baseline covariates and did not explicitly model time-varying adherence or behavior change, part of the observed between-cluster differences in treatment effects may therefore reflect self-selection into higher-engagement patterns, in addition to genuine differences in causal responsiveness.

***The study aligns with ESC/ESH 2023 and ACC/AHA 2024 hypertension guidelines supporting mHealth use and would benefit from future research with better baseline balance, stratified analyses, and harder clinical endpoints (MACE).***

RESPONSE: We thank the reviewer for this insightful, forward-looking comment. We agree that our work is consistent with the direction of recent hypertension guidelines. In line with the reviewer’s suggestion, we have added a brief statement in the Discussion as follows:

Discussion (lines 401-403)

Future studies should evaluate similar approaches in more robust baseline balancing,　stratification analyses, and stricter clinical endpoints such as major cardiovascular adverse events.

***Minor revisions include defining all abbreviations upon first mention, standardizing numerical and p-value formatting, and moving the ChatGPT mention to a Transparency Statement.***

RESPONSE:

Thank you for this helpful comment. We have carefully reviewed the manuscript to ensure that all abbreviations are defined at first mention and that numerical and p value formatting are standardized. In addition, we have moved the description of ChatGPT use from the main text to a brief Transparency statement section, in line with your suggestion.

Transparency statement (lines 460-464)

The authors used ChatGPT only as a writing aid to help refine the English language and improve the clarity of early manuscript drafts. No new scientific content, study design, data analysis, or conclusions were generated by the tool. All text was reviewed, edited, and approved by the authors, who take full responsibility for the final manuscript.

***Overall, the study is relevant and well structured but requires a Major Revision to: (1) acknowledge and quantify statistically significant baseline differences, (2) perform an individual-level analysis to assess the weight of each variable on the final model, and (3) strengthen sensitivity analyses to ensure causal validity. Once these revisions are incorporated, the manuscript will provide robust and clinically meaningful evidence of the personalized benefits of digital interventions for hypertension control.***

RESPONSE: We sincerely appreciate the reviewer’s constructive summary and helpful suggestions. We hope that the revisions detailed above adequately address the reviewer’s concerns and further improve the clarity and robustness of the manuscript.

***Reviewer #5: The study is promising in concept and explores a relevant topic - long-term effectiveness and personalized responses to mHealth hypertension interventions. However, it currently lacks the methodological rigor and transparency required for publication. Some areas of key improvement are to be noted:***

***please provide a clear description of the randomization process and timing. The manuscript claims random allocation but lacks specific details on the randomization process (eg. method of sequence generation, allocation concealment), making it unclear if true randomization occurred.***

RESPONSE: We thank the reviewer for this important comment. We agree that the present study was conducted within the framework of a target trial emulation, and not as an actual randomized trial. The treatment assignment was voluntary and observational, rather than randomized. The term target trial emulation refers to an analytical approach to observational data that aims to emulate the design and analysis of a hypothetical randomized trial, rather than implying that a trial was conducted. Because the two groups differed in several baseline characteristics (e.g., age, BMI, triglycerides, HDL, HbA1c, and Charlson index), we applied G-formula to obtain marginal effect estimates standardized for these covariates, thereby mitigating potential selection bias inherent in nonrandomized data. We have made the following revisions in the revised manuscript:

Methods (lines 86-88)

In this study, we used “target trial emulation” framework, which emulates a target randomized control trial design, combined with a roadmap for causal inference to enhance the quality and interpretation of observational studies.

Table 1.

Individuals were nonrandomly assigned to the treatment arms described below. Randomization was emulated using G-formula, with adjustment for baseline covariates.

Discussion (lines 420-436)]

Fourth, individuals with chronic complications may exhibit lower adherence to both therapeutic regimens and mHealth-based interventions, potentially leading to an underestimation of the observed intervention effects. Fifth, baseline characteristics differed substantially between the treated and untreated groups, raising concerns about the identifiability of causal effects. Although the G-computation approach adjusts for measured confounders through model-based standardization, its validity depends on the positivity assumption. To evaluate this, we examined the empirical distribution of estimated treatment probabilities and observed a limited but clearly non-zero region of overlap between groups (Supplementary Figure 1). This indicates that empirical positivity was restricted but not violated. Finally, the potential for unmeasured confounding remains, despite the application of robust causal inference techniques. In particular, information on medication use, adherence patterns, and socioeconomic or behavioral factors, such as digital literacy and access to technology, was not available and could not be adjusted for. Furthermore, several baseline differences between groups may have partially influenced the observed associations. We addressed this potential confounding using G-computation to obtain marginal estimates adjusted for baseline covariates, and our calculated E-values indicated moderate robustness to unmeasured confounding [46].

***The study design does not fully address the risk of selection bias, especially given the retrospective use of a commercial database and imbalances in baseline characteristics between groups suggest this may be a concern***

***no mention of blinding for participants, assessors, or data analysts, which could introduce bias in outcomes assessment and data interpretation.***

RESPONSE: We thank the reviewer for this important comment. We agree that baseline characteristics differed substantially between the treated and control groups (Table 2), and such imbalance raises valid concerns regarding the identifiability of causal effects. Target trial emulation does not require baseline comparability, but it does require adequate adjustment for confounding and sufficient empirical support for both treatment strategies. In our study, we used the G-formula with a data-adaptive SuperLearner to flexibly model the conditional outcome regression and standardize over the joint distribution of measured covariates. This approach adjusts for baseline differences—such as age, sex, metabolic risk factors, and comorbidities—thereby addressing measured confounding without relying on propensity score weighting.

In response to the reviewer’s concern about the magnitude of imbalance, we further examined the empirical distribution of estimated treatment probabilities (Supplementary Figure 1). Consistent with the selective uptake of the intervention, the treated group demonstrated higher estimated probabilities than the control group, and overall overlap was limited. Nonetheless, the density plot shows a clear non-zero region of common support between approximately 0.02 and 0.06, indicating that empirical positivity was restricted but not violated. We now explicitly acknowledge in the Discussion that both limited overlap and the potential for residual confounding remain inherent limitations of observational analyses.

We also thank the reviewer for the insightful comment regarding potential selection bias. The eligibility criteria were designed to reflect the actual enrollment policy of the mHealth-based intervention program, which targeted adults with common lifestyle-related diseases—including hypertension, diabetes, and dyslipidemia—rather than hypertension alone. These conditions frequently coexist and share overlapping pathophysiological and behavioral risk profiles; thus, the intervention was structured to support comprehensive cardiometabolic risk management rather than a single-disease model. Although the inclusion criteria encompassed multiple diagnoses, hypertension was the prespecified analytic focus, and uncontrolled blood pressure was the sole primary outcome. Including participants with diabetes or dyslipidemia ensured that the study population accurately represented the real-world target population of the mHealth program and the cohort for which the emulated target trial was designed.

We have added and refined the relevant explanations in the revised manuscript accordingly.

Methods (lines 86-88)

In this study, we used “target trial emulation” framework, which emulates a target randomized control trial design, combined with a roadmap for causal inference to enhance the quality and interpretation of observational studies.

Table 1.

Individuals were nonrandomly assigned to the treatment arms described below. Randomization was emulated using G-formula, with adjustment for baseline covariates.

Methods (lines 114-123)

This study included participants diagnosed with hypertension, diabetes, or dyslipidemia who were either receiving medication or had a history of coronary artery disease or stroke. The inclusion criteria reflected the actual enrollment policy of the original mHealth-based intervention program, which targeted adults with common lifestyle-related diseases—including hypertension, diabetes, and dyslipidemia—rather than hypertension alone. These three conditions frequently coexist and share overlapping pathophysiological and behavioral risk factors; therefore, the program was designed to promote comprehensive cardiometabolic risk management rather than a disease-specific approach. Eligible individuals were identified through the Specific Health Checkup and employer-provided medical claims data and were invited to participate in the mHealth-based disease management program.

Discussion (lines 420-436)

Third, while our findings suggest that certain behavioral and metabolic characteristics are associated with intervention effectiveness, the generalizability of these results may be limited to populations with similar demographic and occupational backgrounds. Fourth, individuals with chronic complications may exhibit lower adherence to both therapeutic regimens and mHealth-based interventions, potentially leading to an underestimation of the observed intervention effects. Fifth, baseline characteristics differed substantially between the treated and untreated groups, raising concerns about the identifiability of causal effects. Although the G-computation approach adjusts for measured confounders through model-based standardization, its validity depends on the positivity assumption. To evaluate this, we examined the empirical distribution of estimated treatment probabilities and observed a limited but clearly non-zero region of overlap between groups (Supplementary Figure 1). This indicates that empirical positivity was restricted but not violated. Finally, the potential for unmeasured confounding remains, despite the application of robust causal inference techniques. In particular, information on medication use, adherence patterns, and socioeconomic or behavioral factors, such as digital literacy and access to technology, was not available and could not be adjusted for. Furthermore, several baseline differences between groups may have partially influenced the observed associations. We addressed this potential confounding using G-computation to obtain marginal estimates adjusted for baseline covariates, and our calculated E-values indicated moderate robustness to unmeasured confounding [46].

***4.The statistical methods used are not fully explained and the use of only summary measures (eg. means, medians) without individual data points raises concerns regarding the robustness and reliability of the analysis.***

RESPONSE: We thank the reviewer for this valuable comment. We have carefully revised the Methods and Results sections to provide a clearer and more detailed explanation of the statistical analyses.

Methods (lines 226-232)

Sensitivity analyses

To assess the robustness of the variable selection process, we additionally conducted two types of sensitivity analyses. First, we used alternative thresholds of 0.20 and 0.30 for defining potential effect modifiers. Second, we calculated E-values to evaluate the potential impact of unmeasured confounding, which quantify the minimum strength of association that an unmeasured confounder would need to have with both the exposure and the outcome to fully explain the observed associations [36].

Results (lines 257-264)

For the ATE of the mHealth-based disease management program on uncontrolled hypertension, participation in the program was associated with a 5.2% (95% confidence interval [CI]: 4.4% to 6.0%) lower risk of uncontrolled hypertension, corresponding to a risk ratio of 0.81 (95% CI: 0.78 to 0.84) compared with conventional treatment alone. This absolute risk reduction corresponds to an NNT of 19.2 (95% CI: 16.7 to 22.7) over the study’s follow-up period. The calculated E-value for this association was 1.77. In addition, participation in the program was associated with a mean change in systolic blood pressure of −1.22 mmHg (95% CI: −1.58 to −0.87) and in diastolic blood pressure of −3.67 mmHg (95% CI: −4.14 to −3.22).

Results (lines 281-287)

In the high-benefit cluster, the mean CATE was −0.17 (95% CI: −0.19 to −0.15), while in the low-benefit cluster, the mean CATE was 0.10 (95% CI: 0.08 to 0.12). The corresponding risk ratios were 0.41 (95% CI: 0.34 to 0.48) for the high-benefit cluster and 1.35 (95% CI: 1.28 to 1.42) for the low-benefit cluster. The absolute risk reduction for the high-benefit cluster corresponds to an NNT of 5.9 (95% CI, 5.3 to 6.7) over the study’s follow-up period. The E-values for these estimates were 4.31 and 2.04, respectively.

Discussion (lines 420-436)

Fourth, individuals with chronic complications may exhibit lower adherence to both therapeutic regimens and mHealth-based interventions, potentially leading to an underestimation of the observed intervention effects. Fifth, baseline characteristics differed substantially between the treated and untreated groups, raising concerns about the identifiability of causal effects. Although the G-computation approach adjusts for measured confounders through model-based standardization, its validity depends on the positivity assumption. To evaluate this, we examined the empirical distribution of estimated treatment probabilities and observed a limited but clearly non-zero region of overlap between groups (Supplementary Figure 1). This indicates that empirical positivity was restricted but not violated. Finally, the potential for unmeasured confounding remains, despite the application of robust causal inference techniques. In particular, information on medication use, adherence patterns, and socioeconomic or behavioral factors, such as digital literacy and access to technology, was not available and could not be adjusted for. Furthermore, several baseline differences between groups may have partially influenced the observed associations. We addressed this potential confounding using G-computation to obtain marginal estimates adjusted for baseline covariates, and our calculated E-values indicated moderate robustness to unmeasured confounding [46].

***please provide clear details on ethical approval, consent, or participant privacy consideration***

RESPONSE: Thank you for this helpful comment. Information on institutional ethics approval was already included in the Methods section; however, in the revised manuscript we have created a separate subsection titled “Ethics and data privacy,” where we restate this information and incorporate additional details on consent, anonymization, and data privacy in line with your suggestions.

Methods (lines 234-246)

Ethics and data privacy

This study was approved by the Institute of Transdisciplinary Sciences Ethics Committee, Kanazawa University (Approval number: R6-003). Routinely collected data were linked and anonymized by the program provider (PREVENT, Inc.) in accordance with its data privacy policy and relevant regulations. For participants enrolled in the mHealth-based disease management program, agreement to the in-app privacy policy at registration constituted consent for the use of anonymized program data in research. For individuals who did not enroll in the program, only anonymized health checkup and claims data were made available to the researchers. All data presented were anonymized, and there are no images or other information in this manuscript that could directly identify individual participants.

***please avoid the use of terms like "significant" without providing context in terms of statistical analysis/p-values***

RESPONSE: Thank you for this comment. In the revised manuscript, we carefully checked all uses of the term “significant” and ensured that it is used only when referring to the results of statistical tests. In all other sentences, we removed “significant” and replaced it with neutral descriptive wording.

***the sample imbalance (127 vs 2,965) and lack of adjustment through weighting, matching, etc estimators leave open confounding concerns***

RESPONSE: Thank you very much for your important point-out. In this study, we used the G-formula (also known as the outcome regression approach) to estimate the marginal mean outcome under each treatment strategy. Unlike weighting-based approaches such as inverse probability weighting, the G-formula does not create a reweighted pseudo-population or explicitly balance baseline covariates between treatment groups. Instead, it models the conditional outcome given covariates and then standardizes predictions over the joint distribution of baseline covariates in the full study population. Accordingly, covariate-balance diagnostics such as standardized mean differences are not directly applicable within the G-formula framework.

To address the reviewer’s concern regarding empirical support and comparability, we added Supplementary Figure 1 showing the empirical distribution of estimated treatment probabilities. As expected given the selective uptake of the intervention, the treated group exhibited higher estimated probabilities than the untreated group, resulting in limited overall overlap. Nevertheless, a clear non-zero region of common support was present, indicating that empirical positivity was restricted but not violated. In addition, Table 2 reports baseline characteristics for each group, illustrating the extent of initial imbalance.

We agree that both inverse probability weighting and the G-formula remain susceptible to bias from unmeasured confounding. To evaluate the robustness of our findings to such bias, we calculated E-values. We have incorporated the corresponding clarifications into the Methods, Results, and Discussion sections of the revised manuscript.

Methods (lines 226-232)

Sensitivity analyses

To assess the robustness of the variable selection process, we additionally conducted two types of sensitivity analyses. First, we used alternative thresholds of 0.20 and 0.30 for defining potential effect modifiers. Second, we calculated E-values to evaluate the potential impact of unmeasured confounding, which quantify the minimum strength of association that an unmeasured confounder would need to have with both the exposure and the outcome to fully explain the observed associations [36].

Results (lines 257-264)

For the ATE of the mHealth-based disease management program on uncontrolled hypertension, participation in the program was associated with a 5.2% (95% confidence interval [CI]: 4.4% to 6.0%) lower risk of uncontrolled hypertension, corresponding to a risk ratio of 0.81 (95% CI: 0.78 to 0.84) compared with conventional treatment alone. This absolute risk reduction corresponds to an NNT of 19.2 (95% CI: 16.7 to 22.7) over the study’s follow-up period. The calculated E-value for this association was 1.77. In addition, participation in the program was associated with a mean change in systolic blood pressure of −1.22 mmHg (95% CI: −1.58 to −0.87) and in diastolic blood pressure of −3.67 mmHg (95% CI: −4.14 to −3.22).

Results (lines 281-287)

In the high-benefit cluster, the mean CATE was −0.17 (95% CI: −0.19 to −0.15), while in the low-benefit cluster, the mean CATE was 0.10 (95% CI: 0.08 to 0.12). The corresponding risk ratios were 0.41 (95% CI: 0.34 to 0.48) for the high-benefit cluster and 1.35 (95% CI: 1.28 to 1.42) for the low-benefit cluster. The absolute risk reduction for the high-benefit cluster corresponds to an NNT of 5.9 (95% CI, 5.3 to 6.7) over the study’s follow-up period. The E-values for these estimates were 4.31 and 2.04, respectively.

Discussion (lines 420-436)

Fourth, individuals with chronic complications may exhibit lower adherence to both therapeutic regimens and mHealth-based interventions, potentially leading to an underestimation of the observed intervention effects. Fifth, baseline characteristics differed substantially between the treated and untreated groups, raising concerns about the identifiability of causal effects. Although the G-computation approach adjusts for measured confounders through model-based standardization, its validity depends on the positivity assumption. To evaluate this, we examined the empirical distribution of estimated treatment probabilities and observed a limited but clearly non-zero region of overlap between groups (Supplementary Figure 1). This indicates that empirical positivity was restricted but not violated. Finally, the potential for unmeasured confounding remains, despite the application of robust causal inference techniques. In particular, information on medication use, adherence patterns, and socioeconomic or behavioral factors, such as digital literacy and access to technology, was not available and could not be adjusted for. Furthermore, several baseline differences between groups may have partially influenced the observed associations. We addressed this potential confounding using G-computation to obtain marginal estimates adjusted for baseline covariates, and our calculated E-values indicated moderate robustness to unmeasured confounding [46].

***Include sensitivity analyses using continuous BP outcomes and contextualize results against known clinical thresholds. For eg, the 'absolute risk reduction of 5.2%' lacks clinical context. What does this correspond to in mmHg reduction?***

RESPONSE: We appreciate the reviewer’s insightful comment. In response, we conducted a reanalysis and defined these variables as secondary outcomes, representing the differences in systolic and diastolic blood pressure between baseline and follow-up. Furthermore, we now briefly refer to these secondary results in the Discussion to contextualize the observed 5.2% absolute reduction in uncontrolled hypertension in terms of continuous blood pressure changes. We hope that these additions address the reviewer’s concern.

Methods (lines 186-187)

The secondary outcomes were the differences in systolic and diastolic blood pressure between baseline and follow-up.

Results (lines 262-264)

In addition, participation in the program was associated with a mean change in systolic blood pressure of −1.22 mmHg (95% CI: −1.58 to −0.87) and in diastolic blood pressure of −3.67 mmHg (95% CI: −4.14 to −3.22).

Results (lines 287-293)

Supplementary Tables 1 and 2 present the results of our secondary outcomes, comparing the baseline characteristics of the high-benefit and low-benefit clusters based on the covariates selected in the primary outcome analysis. For the secondary outcome analysis, the mean CATE for systolic blood pressure SBP was −8.34 mmHg (95% CI: −8.85 to −7.83) in the high-benefit cluster and 5.38 mmHg (95% CI: 5.09 to 5.68) in the low-benefit cluster. For diastolic blood pressure DBP, the mean CATE was −11.12 mmHg (95% CI: −11.52 to −10.72) in the high-benefit cluster and 5.52 mmHg (95% CI: 5.14 to 5.90) in the low-benefit cluster.

Discussion (lines 316-327)

While minimal clinically important differences for blood pressure reduction in digital interventions remain unclear, our study employed a pragmatic trial design using uncontrolled hypertension—defined as systolic ≥140 mmHg or diastolic ≥90 mmHg—as a clinically meaningful endpoint. Based on this definition, participation in the mHealth-based program resulted in a 5.2% absolute reduction in the prevalence of uncontrolled hypertension compared with conventional treatment. This approach enhances the real-world applicability of our findings and aligns the evaluation of digital interventions with established clinical thresholds. Consistent with this threshold-based endpoint, secondary analyses of continuous blood pressure values showed mean changes in systolic and diastolic blood pressure (−1.22 and −3.67 mmHg, respectively). These mean reductions are compatible with the observed decrease in uncontrolled hypertension and should be interpreted alongside the substantial heterogeneity in individual treatment responses described below.

Discussion (lines 388-396)

A key strength of this study lies in its use of a three-step, data-adaptive framework that integrates outcome regression via the G-formula with SuperLearner-based model specification to estimate treatment heterogeneity. By combining the G-formula with a SuperLearner ensemble model, we can assess individual-level treatment effects while minimizing overfitting and preserving interpretability. This approach extends beyond traditional ATE estimates, providing a more nuanced understanding of how different subgroups respond to mHealth-based interventions [38]. Moreover, the consistent direction of effects between the primary binary endpoint and the secondary continuous outcomes supports the robustness of the estimated heterogeneous treatment effects.

We hope that the revisions and clarifications sufficiently address the concerns raised and improve the clarity and quality of our manuscript. We appreciate your time and consideration, and we look forward to your further evaluation.
