# Supplementary material for: Heterogeneous associations of a mobile health-based disease management program on uncontrolled hypertension: A target trial emulation study
Source: PLOS Digit Health. 2026 Mar 5;5(3):e0001268. doi: 10.1371/journal.pdig.0001268 (PMC12962524; doi:10.1371/journal.pdig.0001268)
Supplement: Attachment — Submitted filename: Mystar_TTE_Response_R2.docx [file pdig.0001268.s008.docx]

**Response to Reviewers**

We sincerely thank the Reviewers and for their thoughtful and constructive feedback. We have carefully revised our manuscript in accordance with the comments provided. Below we provide point-by-point responses to each comment. All modifications to the manuscript are highlighted and noted with track changes.

**Review Comments to the Author**

***Reviewer #4: This study estimates the long-term effectiveness of a chronic disease management program based on mHealth for patients with uncontrolled hypertension, a significant pathology with a high burden of morbidity and mortality. A target trial emulation design was chosen, employing causal inference methods and machine learning as an appropriate approach. The topic is highly relevant in the fields of medicine and digital medicine, as it addresses the need for even stronger evidence regarding the sustainability of mHealth interventions in real-world populations and their monitoring. In general, the study is well-structured, methodologically ambitious, and effectively utilizes complex and preferred analytical tools, which strengthens its results, particularly from a methodological standpoint.

The study presents significant structural limitations that must be considered when interpreting the results. The main one is the overall imbalance between the treatment groups, as well as the lack of control over fundamental variables that have an impact, since these in themselves represent well-studied risk factors such as sex, metabolic profile, and comorbidity burden. This generates a significant risk of bias when interpreting the results. Although the authors adequately correct for these biases using the G-formula, showing that positivity is not strongly violated, the limited overlap between groups restricts causal inference at the individual level. Stratified studies should be conducted due to the baseline differences between the groups. We must consider that, although there is no statistical impact, the aforementioned factors are considered individual risk factors in classic, highly standardized studies with rigorous methodology. Added to this is the potential for uncontrolled confounding, especially variables associated with the number and type of antihypertensive medications, medication adherence, socioeconomic status, digital literacy, and actual use of the application. These considerations are considered particularly important for interpreting heterogeneity analyses, since the benefit found may be contaminated by self-selection and greater compliance capacity, rather than causal differences in program response.***

***We consider the following recommendations for the authors: this study could be further enriched by including longitudinal measures of participation and application use, which would allow for a better separation of the program's causal effect from the adherence-mediated effect—that is, an analysis of adherence. In addition, the aforementioned analyses stratified by sex or pre-existing cardiovascular disease, as well as by other variables, should be included. We consider it important to emphasize that individual treatment effects should be considered as prioritization criteria and aids in clinical decision-making, but never as criteria for excluding patients. From an editorial perspective, the manuscript is enhanced by defining itself as a methodological contribution to improving how digital interventions are evaluated using real-world data, rather than a demonstration of causal efficacy.

The above study is well-designed, innovative, and aligns with current hypertension guidelines that advocate for the complementary use of digital tools to achieve treatment and control. Its results should be interpreted with caution, given the inherent limitations of an observational design, but it can serve as a starting point for developing more personalized digital health strategies. With the necessary corrections and maintaining an interpretive tone, the manuscript is ready for publication.***

RESPONSE:

Thank you very much for this comprehensive and thoughtful comment. We fully agree with your view that the structural imbalance between treatment groups, limited overlap in baseline characteristics, and potential residual confounding require a cautious interpretation of our findings, particularly regarding heterogeneous treatment effects.

After careful consideration, we have expanded the Limitations section of the Discussion to (i) more explicitly acknowledge that the observed heterogeneity may partly reflect self-selection mechanisms and differences in engagement or adherence, (ii) note that restricted empirical positivity constrains individual-level causal interpretation, and (iii) clarify that our ITE estimates are intended to support clinical decision-making and prioritization rather than to define eligibility or exclusion criteria for digital health interventions. We also now emphasize that the main contribution of this work is methodological, illustrating how target trial emulation and data-adaptive modeling can be applied to real-world data, rather than establishing definitive causal efficacy. We hope these revisions address the reviewer’s concerns and make the interpretive boundaries of our study clearer to readers.

Discussion (lines 412-416)

Fourth, individuals with chronic complications may exhibit lower adherence to both therapeutic regimens and mHealth-based interventions, potentially leading to an underestimation of the observed intervention effects. Moreover, observed heterogeneity may partially reflect self-selection mechanisms and differential capacity for engagement or adherence, rather than true causal effect modification.

Discussion (lines 417-425)

Fifth, baseline characteristics differed substantially between the treated and untreated groups, raising concerns about the identifiability of causal effects. Although the G-computation approach adjusts for measured confounders through model-based standardization, its validity depends on the positivity assumption. To evaluate this, we examined the empirical distribution of estimated treatment probabilities and observed a limited but clearly non-zero region of overlap between groups (Supplementary Figure 1). While empirical positivity was restricted but not violated, this limited overlap constrains individual-level causal interpretation, particularly for heterogeneous treatment effect estimation.

Discussion (lines 433-438)

The ITE estimates should therefore be used to support clinical decision-making and prioritization, not to inform eligibility or exclusion rules for digital health interventions. Accordingly, our findings are best interpreted as illustrating how target trial emulation can be applied to real-world data to evaluate mHealth-based disease management programs, while providing supportive evidence that such programs may be associated with better blood pressure control in routine care.

We hope that the revisions and clarifications sufficiently address the concerns raised and improve the clarity and quality of our manuscript. We appreciate your time and consideration, and we look forward to your further evaluation.
