# Supplementary material for: Heterogeneous associations of a mobile health-based disease management program on uncontrolled hypertension: A target trial emulation study
Source: PLOS Digit Health. 2026 Mar 5;5(3):e0001268. doi: 10.1371/journal.pdig.0001268 (PMC12962524; doi:10.1371/journal.pdig.0001268)
Supplement: Attachment — Submitted filename: Mystar_TTE_Response_R3.docx [file pdig.0001268.s009.docx]

**Response to Reviewers**

We sincerely thank the Reviewers and for their thoughtful and constructive feedback. We have carefully revised our manuscript in accordance with the comments provided. Below we provide point-by-point responses to each comment. All modifications to the manuscript are highlighted and noted with track changes.

**Review Comments to the Author**

***Reviewer #4: I appreciate how carefully and thoughtfully the authors revised the text. The changes make it much clearer and set clear limits on how the data can be interpreted. The longer discussion of baseline imbalance, limited empirical positivity, and possible self-selection and adherence-related mechanisms does a good job of addressing the primary methodological issues that were brought up before.

At this point, I would want to explain in more detail why I suggested changing the title of the manuscript. The work utilizes sophisticated causal inference methodologies, such as target trial emulation, G-computation, and machine learning–based outcome modeling; nonetheless, the foundational data are inherently observational. Consequently, the study is fundamentally constrained in its capacity to directly assess the causal effect of hypertension therapy. Essential aspects of clinical management—including treatment intensification, medication adherence, clinician decision-making, and longitudinal variations in therapy—cannot be entirely observed or regulated within the existing paradigm. As a result, residual confounding and selection mechanisms cannot be completely eliminated, even with strong analytical techniques.

From a methodological standpoint, the results endorse inferences concerning modeled treatment effects and relationships within a causal framework, rather than conclusive causal determinations regarding the efficacy of hypertension care. The primary value of the manuscript is its demonstration of how contemporary causal and data-adaptive methodologies can be utilized to investigate variation in blood pressure outcomes and to guide hypothesis formulation, prioritization, and the design of future studies. However, the existing title might make some readers think that there is a level of causal certainty that an observational study, no matter how rigorous, can't fully support.

For this reason, I think that changing the title is a fair and helpful way to keep the scientific value of the work while making sure that readers understand what the study can really tell them. Putting the words "evaluation," "association," or "methodological assessment" in the title would better show what the study can and can't say, without making it less useful for clinical practice or digital health research.

I advise using titles that focus on evaluation, association, or methodological assessment instead of direct causal consequences of hypertension management to better match the manuscript title with the observational study design and its inferential scope. Some other options are:

Assessment of a mobile health-oriented disease management program for uncontrolled hypertension by target trial emulation.

Blood pressure regulation linked to a mobile health-oriented illness management program: a practical target trial emulation study

Diversity in blood pressure outcomes among participants in a mobile health–based hypertension management program
All of these choices keep the study's scientific contribution and methodological rigor while also making it clear that the publication does not directly quantify the causal effect of managing hypertension, which cannot be fully demonstrated with the current observational design.***

RESPONSE:

We thank the reviewer for this helpful suggestion. We agree that the original title could be interpreted as implying a level of causal certainty that is not fully supported by an observational design. Accordingly, we have revised the title to emphasize association and the target trial emulation framework, and we made corresponding minor edits to the Abstract and Introduction. While we appreciate the alternative title options provided, we chose wording that is consistent with the terminology used throughout the revised manuscript to ensure clarity and internal coherence.

Title: Heterogeneous associations of a mobile health-based disease management program with uncontrolled hypertension: a target trial emulation study

Abstract (lines 21-24)

This study examined the association of a mobile health-based disease management program for uncontrolled hypertension and assessed treatment effect heterogeneity using a target trial emulation framework.

Introduction (lines 79-82)

To address these gaps in knowledge, this study examined the heterogeneity in the association between of an mHealth-based disease management program [23,24] and uncontrolled hypertension over a one-year follow-up using the target trial emulation framework.

We hope that the revisions and clarifications sufficiently address the concerns raised and improve the clarity and quality of our manuscript. We appreciate your time and consideration, and we look forward to your further evaluation.
